# Association between end-tidal sevoflurane concentration and postoperative cognitive dysfunction and pain sensitivity in elderly patients under general anesthesia

**DOI:** 10.3389/fnagi.2026.1761052

**Published:** 2026-02-16

**Authors:** Yunyun Zhou, Han Zheng, Zhengyu Li, Jiaxuan Wang, Xue Zhang, Lifen Zheng, Hong Luo, Heng Yang

**Affiliations:** 1 Department of Anesthesiology, The Third Affiliated Hospital of Anhui Medical University (First People’s Hospital of Hefei), Hefei, Anhui, China; 2 Department of Pain, The First Affiliated Hospital of Zhejiang Chinese Medical University (Zhejiang Provincial Hospital of Traditional Chinese Medicine), Hangzhou, Zhejiang, China; 3 Department of Anesthesiology, The First Affiliated Hospital of Anhui Medical University, Hefei, Anhui, China

**Keywords:** cognitive function, elderly, general anesthesia, pain sensitivity, sevoflurane concentration

## Abstract

**Objective:**

To analyze the association between end-tidal sevoflurane concentration and postoperative cognitive dysfunction (POCD) and pain sensitivity in elderly patients under general anesthesia.

**Methods:**

A total of 121 elderly patients undergoing abdominal surgery were enrolled and divided into a low-concentration group (0.8–1.2 MAC, *n* = 61) and a high-concentration group (1.5–2.0 MAC, *n* = 60). End-tidal sevoflurane concentration, recovery parameters, Mini-Mental State Examination (MMSE) scores, POCD incidence, pain sensitivity (PSQ score, mechanical hypersensitivity areas and thresholds), and serum levels of neurological markers (NSE, ApoJ, NGF) and pain mediators (PGE2, 5-HT) were compared between groups. Pearson correlation and multivariate logistic regression were used to assess associations and influencing factors for POCD.

**Results:**

The high-concentration group had longer recovery times but a lower incidence of POCD (10.00% vs. 24.59%) and higher MMSE scores on postoperative days 1 and 3 (*P* < 0.05). Pain sensitivity measures were reduced in the high-concentration group on postoperative day 1, with lower PSQ scores, smaller mechanical hypersensitivity areas, and higher mechanical thresholds (*P* < 0.05). Compared to the low-concentration group, the high-concentration group exhibited significantly lower postoperative levels of NSE, ApoJ, PGE2, and 5-HT, and higher NGF levels (*P* < 0.05). End-tidal sevoflurane concentration positively correlated with MMSE scores, mechanical thresholds, and NGF levels, and negatively correlated with pain sensitivity measures, NSE, ApoJ, PGE2, and 5-HT (*P* < 0.05). Multivariate analysis identified age and preoperative elevations of NSE, ApoJ, PGE2, and 5-HT as risk factors for POCD, while higher sevoflurane concentration and preoperative NGF levels were protective factors (*P* < 0.05).

**Conclusion:**

Higher end-tidal sevoflurane concentration is associated with improved postoperative cognitive function and reduced pain sensitivity in elderly patients, likely mediated by the modulation of neurological and pain-related biomarkers, serving as a protective factor against POCD.

## 1 Introduction

General anesthesia is a common anesthetic protocol for abdominal surgery, rendering patients pain-free and providing optimal conditions for surgical procedures. However, due to diminished organ compensatory capacity, reduced physiological reserve, and decreased tolerance in elderly patients, combined with the effects of anesthetic agents themselves, surgical trauma, and stress response, they are prone to a series of postoperative complications, among which Postoperative Cognitive Dysfunction (POCD) is the most prevalent (Kitthanyateerakul et al., 2024; Viderman et al., 2023). The occurrence of POCD not only prolongs the patient’s recovery process and increases medical costs but also reduces their postoperative quality of life, adversely affects prognosis, and imposes a substantial burden on families and society (Suraarunsumrit et al., 2024). Sevoflurane is a modern inhalational anesthetic agent. It produces sedative, hypnotic, and muscle relaxant effects by interfering with signal transduction at the neuromuscular junction, inhibiting the central nervous system and excitatory transmission between nerve cells, thereby reducing the brain’s responsiveness to external stimuli (Xu et al., 2013; Zhu et al., 2023). Owing to its advantages such as rapid induction, fast metabolism, and smooth emergence, sevoflurane is widely used for anesthetic maintenance in elderly patients.

As a significant stressor, postoperative pain can activate the hypothalamic-pituitary-adrenal axis, thereby triggering a rise in cortisol that promotes neuronal atrophy and apoptosis—processes that ultimately impair cognitive function (Cao et al., 2023). Pain sensitivity reflects an individual’s tolerance and response to noxious stimuli and serves as a crucial indicator for assessing anesthetic quality and postoperative outcomes. Studies have confirmed that the perioperative use of agents such as sevoflurane may alter postoperative pain sensitivity by influencing central sensitization processes. Furthermore, changes in pain sensitivity can subsequently impact cognitive function via neuroendocrine stress responses (Zeng et al., 2023). Currently, clinical research quantitatively elucidating the relationship between sevoflurane inhalation concentration and cognitive function and pain sensitivity, particularly in high-risk populations like elderly patients undergoing abdominal surgery, remains insufficient (Laferrière-Langlois et al., 2024). Some studies support the conclusion that sevoflurane inhalation may cause potential damage to the central nervous system through various mechanisms, including inducing neuroinflammation, modulating neurotransmitters, and promoting ββ-amyloid protein deposition (Yang et al., 2024). Conversely, other research indicates that sevoflurane helps maintain cerebral oxygen balance during cardiopulmonary bypass and ameliorates cognitive impairment in a dose-dependent manner (Hu et al., 2014). Therefore, this study aims to analyze the relationship between end-tidal sevoflurane concentration during general anesthesia and postoperative cognitive function and pain sensitivity in elderly patients, with the goal of informing the optimization of sevoflurane anesthesia protocols for this population and reducing the risk of POCD.

## 2 Materials and methods

### 2.1 General data

A cohort consisting of 121 elderly patients was included in this study, all of whom underwent abdominal surgery between February 2023 and January 2025. According to the end-tidal sevoflurane concentration maintained during general anesthesia, they were allocated into a low-concentration group (0.8–1.2 MAC, *n* = 61) and a high-concentration group (1.5–2.0 MAC, *n* = 60). The low-concentration group comprised 32 males and 29 females, aged 60–84 years with a mean age of 70.15 ± 5.18 years. Their BMI ranged from 19.25 to 27.45 kg/m^2^, with a mean of 23.48 ± 2.48 kg/m^2^. According to the ASA physical status classification (Apfelbaum and Connis, 2019), 17 patients were grade I and 44 were grade II. Types of surgery included: 25 gastric surgeries, 21 colorectal surgeries, and 15 hepatobiliary surgeries. Comorbidities included hypertension in 27 patients and diabetes mellitus in 24 patients. The high-concentration group included 30 males and 30 females, aged 62–85 years with a mean age of 71.13 ± 4.89 years. Their BMI ranged from 19.10 to 27.89 kg/m^2^, with a mean of 23.24 ± 2.52 kg/m^2^. ASA classification included 15 grade I and 45 grade II patients. Types of surgery included: 23 gastric surgeries, 23 colorectal surgeries, and 14 hepatobiliary surgeries. Comorbidities included hypertension in 30 patients and diabetes mellitus in 24 patients. Comparability was confirmed between the groups, with no statistically significant disparities observed in any of the baseline characteristics (*P* > 0.05).

### 2.2 Eligibility criteria

(1) Inclusion criteria: ➀ Age 60–85 years; ➁ ASA physical status classification of I or II; ➂ Patients or their family members voluntarily provided written informed consent; ➃ Normal preoperative pain sensation and cognitive function, clear consciousness. (2) Exclusion criteria: ➀ Allergy to sevoflurane or other medications used in the study; ➁ Severe hepatic or renal insufficiency; ➂ Patients undergoing emergency or secondary surgery; ➃ History of chronic alcohol abuse or substance abuse; ➄ Preoperative comorbid psychiatric disorders or POCD; ➅ Severe visual or auditory impairment preventing cooperation with relevant assessments; ➆ History of long-term alcohol use; ➇ Long-term use of medications affecting neuropsychiatric function, such as anxiolytics, hypnotics, narcotic analgesics, antidepressants, etc.; ➈ History of previous abdominal surgery. (3) Elimination criteria: ➀ Development of severe postoperative complications, such as multiple organ failure or major hemorrhage, affecting cognitive function assessment; ➁ Intraoperative conversion to a different anesthetic regimen; ➂ Requiring reoperation postoperatively; ➃ Intraoperative BIS values persistently failed to reach the target range despite adjustment.

### 2.3 Anesthetic technique

Following their arrival in the operating suite, an IV line was placed in a patient’s arm to administer fluids. Routine monitoring of vital signs, including peripheral SpO_2_, ECG, and MAP, was initiated. Anesthesia Induction: Intravenous administration of midazolam (0.03 mg/kg), sufentanil (0.3–0.5 μg/kg), propofol (1.5–2.0 mg/kg), and cisatracurium (0.15–0.20 mg/kg). Following successful induction of anesthesia, endotracheal intubation was performed. Ventilatory support was commenced using an anesthesia machine, with the initial parameters set as outlined: Respiratory rate set at 12–16 breaths/min, tidal volume at 8 mL/kg, fresh gas flow at 2 L/min, and inspired oxygen concentration (FiO2) at 1.0. End-tidal carbon dioxide partial pressure (PetCO_2_) was maintained between 35 and 45 mmHg. Anesthesia Maintenance: The low-concentration group received inhaled sevoflurane at 0.8–1.2 minimum alveolar concentration (MAC), while the high-concentration group received 1.5–2.0 MAC sevoflurane. Concurrently, both groups received a continuous infusion of remifentanil at 0.1–0.2 μg/(kg⋅min) to maintain a Bispectral Index (BIS) value between 40 and 60. BIS monitoring was performed using a Philips MP50 monitor. Calibration was strictly completed according to the device manual prior to surgery, and data recording and interpretation were managed by an anesthesiologist with specialized training. Adjustment of anesthesia depth was based on a comprehensive assessment combining BIS (target 40–60), MAP fluctuations (changes not exceeding ± 20% of baseline), and heart rate changes to ensure adequate sedation depth. If a patient in the low-concentration group, after receiving the maximum dose of sevoflurane (1.2 MAC) and having remifentanil adjusted to its maximum rate [0.2 μg/(kg⋅min)], still exhibited a BIS value > 60 for more than 5 min (after excluding equipment malfunction), they were considered to have insufficient sedation and were excluded from the group. If the BIS value was < 40, the sevoflurane concentration and/or remifentanil infusion rate were reduced until BIS returned to the target range. Intraoperative muscle relaxation was maintained with intermittent intravenous boluses of cisatracurium (0.03 mg/kg). The muscle relaxant was discontinued 30 min before the anticipated end of surgery, remifentanil was stopped 10 min preoperatively, and sevoflurane inhalation was terminated at the conclusion of the surgery. Postoperative pain was managed with a Patient-Controlled Intravenous Analgesia (PCIA) regimen containing sufentanil (1.5 μg/kg) and tropisetron (10 mg), constituted in 100 mL of normal saline. The pump was configured with the following settings: A basal rate of 2 mL/h; 0.5 mL bolus doses; and a lockout period of 15 min.

### 2.4 Observation indicators

(1) Quality of postoperative recovery. The following parameters were recorded: end-tidal sevoflurane concentration, emergence time, time to orientation recovery, spontaneous breathing recovery time, extubation time, and the total dosage of remifentanil were recorded. (2) Cognitive function. Cognitive function was evaluated preoperatively on day 1 and postoperatively on days 1, 3, and 7 using the MMSE (Li et al., 2016). This scale evaluates language ability, recall, orientation, attention, and calculation skills, with a maximum score of 30; cognitive function is positively correlated with the score. Postoperative Cognitive Dysfunction (POCD) was defined as a decrease in the MMSE score of ≥ 2 points from the preoperative value. (3) Pain Sensitivity. On postoperative day 1, pain sensitivity was evaluated using the Pain Sensitivity Questionnaire (PSQ) (Quan et al., 2018). This questionnaire comprises 17 items, each scored from 0 to 10; the total PSQ score is the average of all item scores. Additionally, the medial arm mechanical hypersensitivity area (on the medial aspect of the dominant forearm) and the incisional mechanical hypersensitivity area (within a 5 cm radius of the incision) as well as their corresponding mechanical hypersensitivity thresholds were measured on postoperative day 1 using a Von Frey algesimeter manufactured by North Coast Medical, Inc., United States. (4) Neurological Damage Markers and Pain Mediators. Following an overnight fast, 5 mL of venous blood was collected from each patient at two time points: the mornings of the day before and the day after the operation. After centrifugation (centrifugal radius: 6 cm; speed: 3,000 rpm) for 10 min, the supernatant was collected. Serum levels of Nerve Growth Factor (NGF), Neuron-Specific Enolase (NSE), Apolipoprotein J (ApoJ), 5-Hydroxytryptamine (5-HT), and Prostaglandin E2 (PGE2) were measured using enzyme-linked immunosorbent assay (ELISA). All procedures were strictly performed according to the manufacturer’s instructions. (5) Analysis of Factors Influencing POCD. Clinical data (age, gender, BMI, history of hypertension, history of diabetes, ASA classification), surgery-related data (sevoflurane concentration, type of surgery, duration of anesthesia), and laboratory indicators (baseline serum levels of NSE, ApoJ, NGF, PGE2, and 5-HT) were compared between the POCD and non-POCD groups. A multivariate logistic regression model was utilized to examine the factors associated with POCD occurrence in elderly patients post-general anesthesia.

### 2.5 Statistical methods

Statistical analysis was performed using SPSS software (version 25.0). Measurement data were tested for normality. Normally distributed measurement data are presented as the mean ± standard deviation ($\bar{\chi }$ ± s) and were compared using the independent samples *t*-test. Categorical data are expressed as number (percentage) [n (%)] and were compared using the chi-square (χ^2^) test. Pearson correlation analysis was conducted to examine the relationship between end-tidal sevoflurane concentration and various indicators. A multivariate logistic regression model was established to identify factors influencing the occurrence of POCD in elderly patients after general anesthesia. Statistical significance was established using a criterion of *P* < 0.05.

## 3 Results

### 3.1 Comparison of end-expiratory sevoflurane concentrations, postoperative recovery quality, and pain sensitivity between the two groups

Two patients in the low-concentration group exhibited transient BIS > 60 (duration < 5 min), which normalized after adjustment, and they were not excluded; no patients experienced persistent under- or over-sedation. The high-concentration group exhibited longer emergence time, time to orientation recovery, spontaneous breathing recovery time, and extubation time compared to the low-concentration group. The end-tidal sevoflurane concentration was higher in the high-concentration group (*P* < 0.05). On postoperative day 1, the PSQ score and the medial arm and incisional mechanical hypersensitivity areas in the high-concentration group were significantly lower than those in the low-concentration group, while the medial arm and incisional mechanical hypersensitivity thresholds were significantly higher (*P* < 0.05). There was no statistically significant difference in total remifentanil dosage between the two groups (*P* > 0.05) (see Table 1).

**TABLE 1:** Comparison of end-expiratory sevoflurane concentrations, postoperative recovery quality, and pain sensitivity between the two groups ($\bar{\chi }$ ± s).

Group	End-tidal sevoflurane concentration (MAC)	Emergence time (min)	Time to orientation recovery (min)	Spontaneous breathing recovery Time (min)	Extubation time (min)	Remifentanil dosage [μ g/(kg⋅min)]	PSQ score (scores)	Mechanical hypersensitivity area (cm^2^)		Mechanical hypersensitivity threshold (g)	
								Medial arm	Incision	Medial arm	Incision
Low-concentration group (*n* = 61)	0.95 ± 0.15	12.51 ± 3.28	14.15 ± 3.85	10.15 ± 1.85	12.67 ± 3.05	0.15 ± 0.03	5.74 ± 1.03	64.02 ± 6.32	64.58 ± 5.89	41.26 ± 5.19	40.95 ± 4.26
High-concentration group (*n* = 60)	1.25 ± 0.20	16.47 ± 4.02	19.47 ± 4.02	13.65 ± 2.99	16.15 ± 3.18	0.14 ± 0.04	4.63 ± 0.86	53.15 ± 6.05	55.18 ± 4.98	51.16 ± 4.68	52.26 ± 4.68
t	8.977	5.942	7.435	7.757	6.144	1.557	6.383	9.670	9.483	11.002	13.920
*P*	< 0.001	< 0.001	< 0.001	< 0.001	< 0.001	0.122	< 0.001	< 0.001	< 0.001	< 0.001	< 0.001

### 3.2 Comparison of MMSE scores at different time points and POCD incidence between the two groups

**FIGURE 1:**
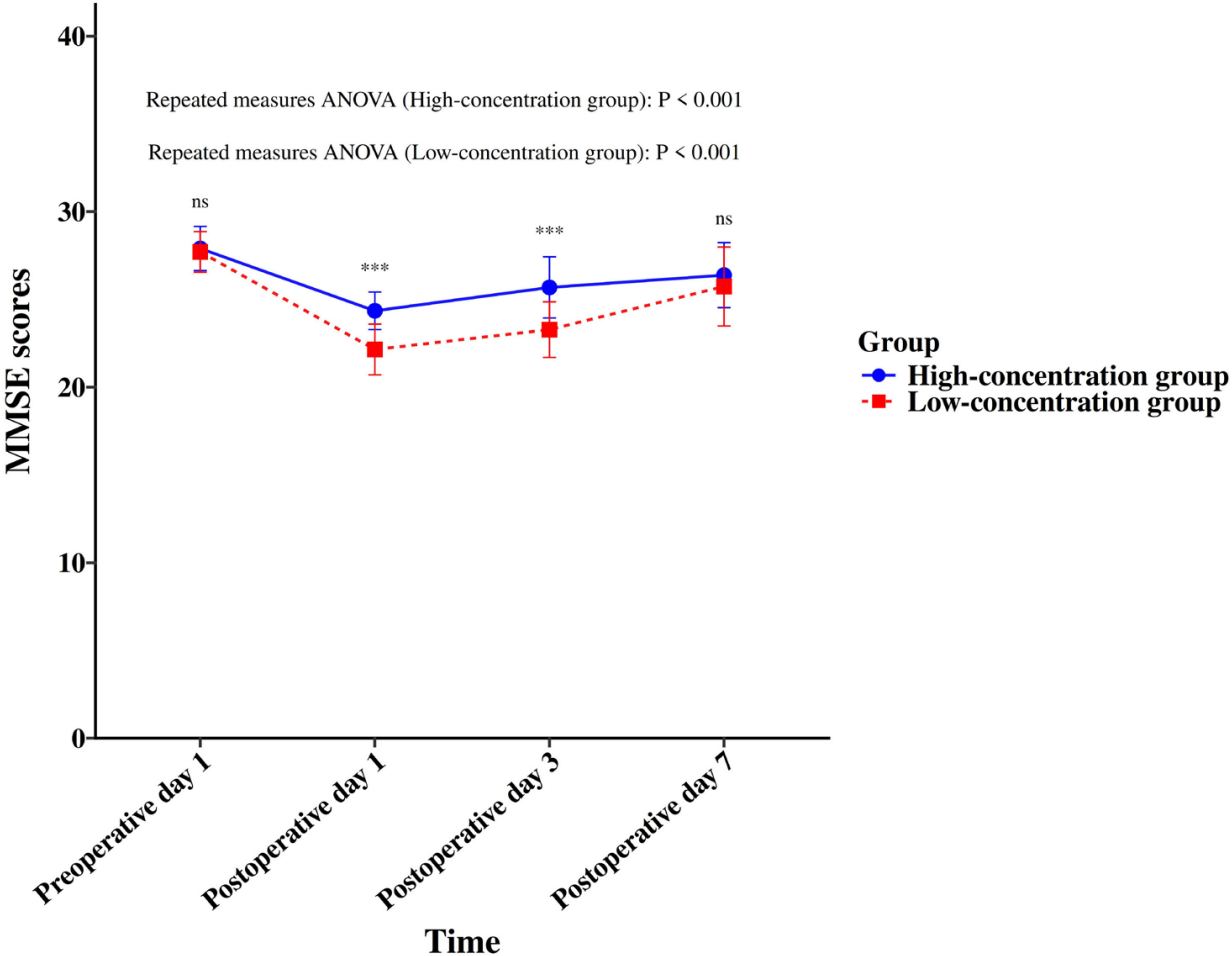
Serial MMSE scores: a comparison between the two groups. ***p < 0.001.

The incidence of POCD in the high-concentration group was 10.00% (6/60), which was significantly lower than the 24.59% (15/61) observed in the low-concentration group (χ^2^ = 4.489, *P* = 0.034). Although MMSE scores were comparable between the two groups preoperatively and on postoperative day 7 (*P* > 0.05), the high-concentration group demonstrated significantly superior cognitive performance, as indicated by higher MMSE scores, on both postoperative days 1 and 3 (*P* < 0.05) (see Figure 1).

### 3.3 Comparison of neurotoxic factors and pain mediators before and after surgery between two groups

Compared with their respective preoperative day 1 levels, serum NSE, ApoJ, PGE2, and 5-HT levels increased, while NGF levels decreased on postoperative day 1 in both groups (*P* < 0.05). Compared with the low-concentration group, the high-concentration group exhibited significantly reduced serum NSE ApoJ, PGE2, and 5-HT levels on postoperative day 1, while displaying a marked elevation in NGF levels (*P* < 0.05) (see Table 2).

**TABLE 2:** Comparison of neurotoxic factors and pain mediators before and after surgery between two groups ($\bar{\chi }$ ± s).

Group	NSE (μ g/L)		ApoJ (mg/L)		NGF (μ g/L)		PGE2 (pg/mL)		5-HT (ng/mL)	
	Preoperative day 1	Postoperative day 1	Preoperative day 1	Postoperative day 1	Preoperative day 1	Postoperative day 1	Preoperative day 1	Postoperative day 1	Preoperative day 1	Postoperative day 1
Low-concentration group (*n* = 61)	4.05 ± 0.69	8.25 ± 1.84***	120.32 ± 13.25	145.84 ± 15.63***	403.36 ± 38.49	296.65 ± 22.45***	86.26 ± 10.25	168.26 ± 15.26***	124.26 ± 15.26	200.65 ± 21.74***
High-concentration group (*n* = 60)	4.12 ± 0.76	6.99 ± 1.15***	119.58 ± 14.36	129.65 ± 18.54**	400.78 ± 39.85	331.19 ± 25.87***	85.79 ± 9.68	136.85 ± 16.02***	122.98 ± 14.68	173.53 ± 19.54***
t	0.531	4.505	0.295	5.198	0.362	7.844	0.259	11.051	0.470	7.213
P	0.597	< 0.001	0.769	< 0.001	0.718	< 0.001	0.796	< 0.001	0.639	< 0.001

Compared with Preoperative Day 1 within the same group, **P < 0.01, ***P < 0.001.

### 3.4 Correlation between end-tidal sevoflurane concentration and MMSE score, pain sensitivity, neurological damage markers, and pain mediators on postoperative day 1

**FIGURE 2:**
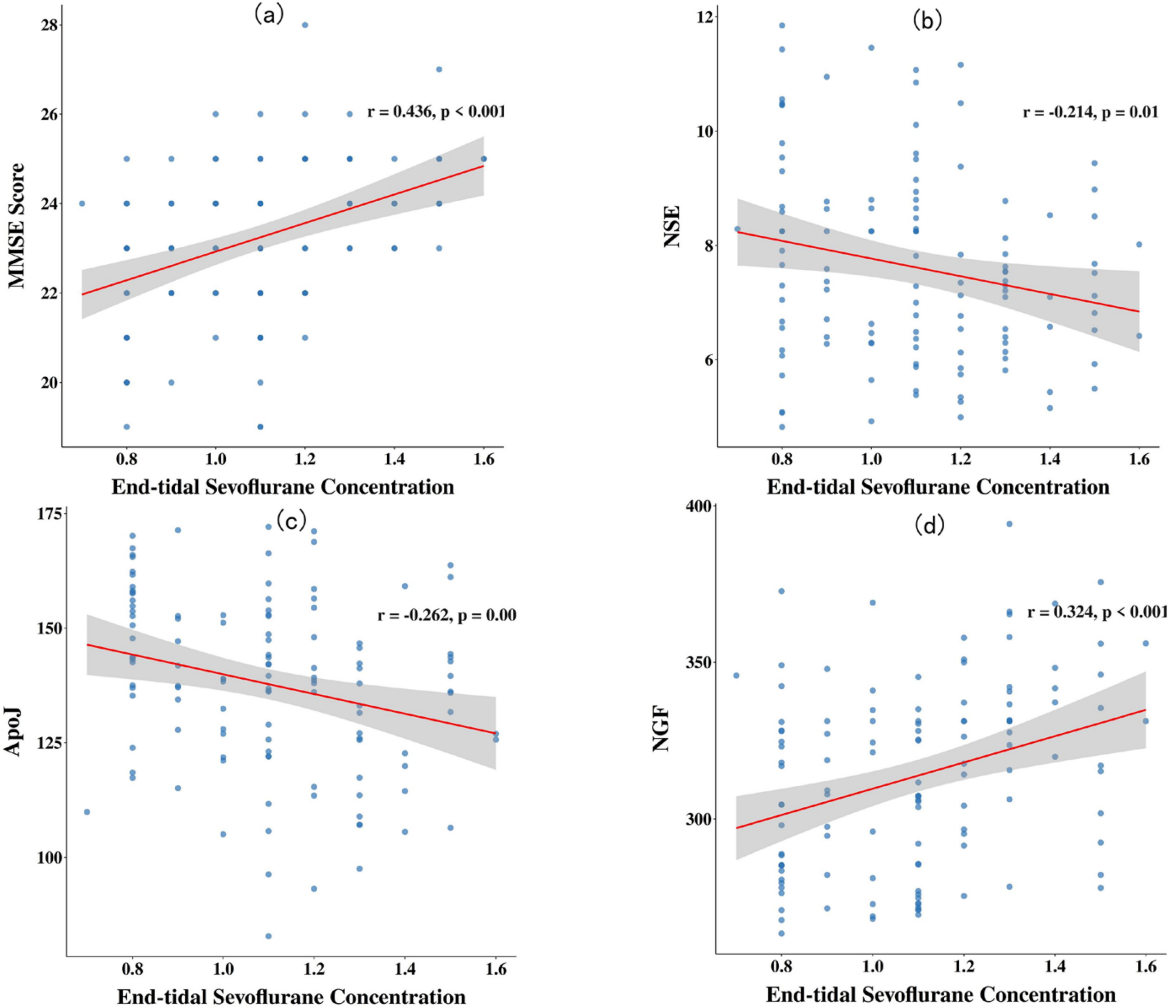
Scatter plots of correlations between end-tidal sevoflurane concentration and cognitive function/neurological damage markers. **(a)** End-tidal sevoflurane concentration was positively correlated with the MMSE score on postoperative day 1 (*r* = 0.436, *P* < 0.001); **(b)** end-tidal sevoflurane concentration was negatively correlated with the serum NSE level on postoperative day 1 (*r* = -0.214, *P* = 0.018); **(c)** end-tidal sevoflurane concentration was negatively correlated with the serum ApoJ level on postoperative day 1 (*r* = -0.262, *P* = 0.004); **(d)** end-tidal sevoflurane concentration was positively correlated with the serum NGF level on postoperative day 1 (*r* = 0.324, *P* < 0.001).

**FIGURE 3:**
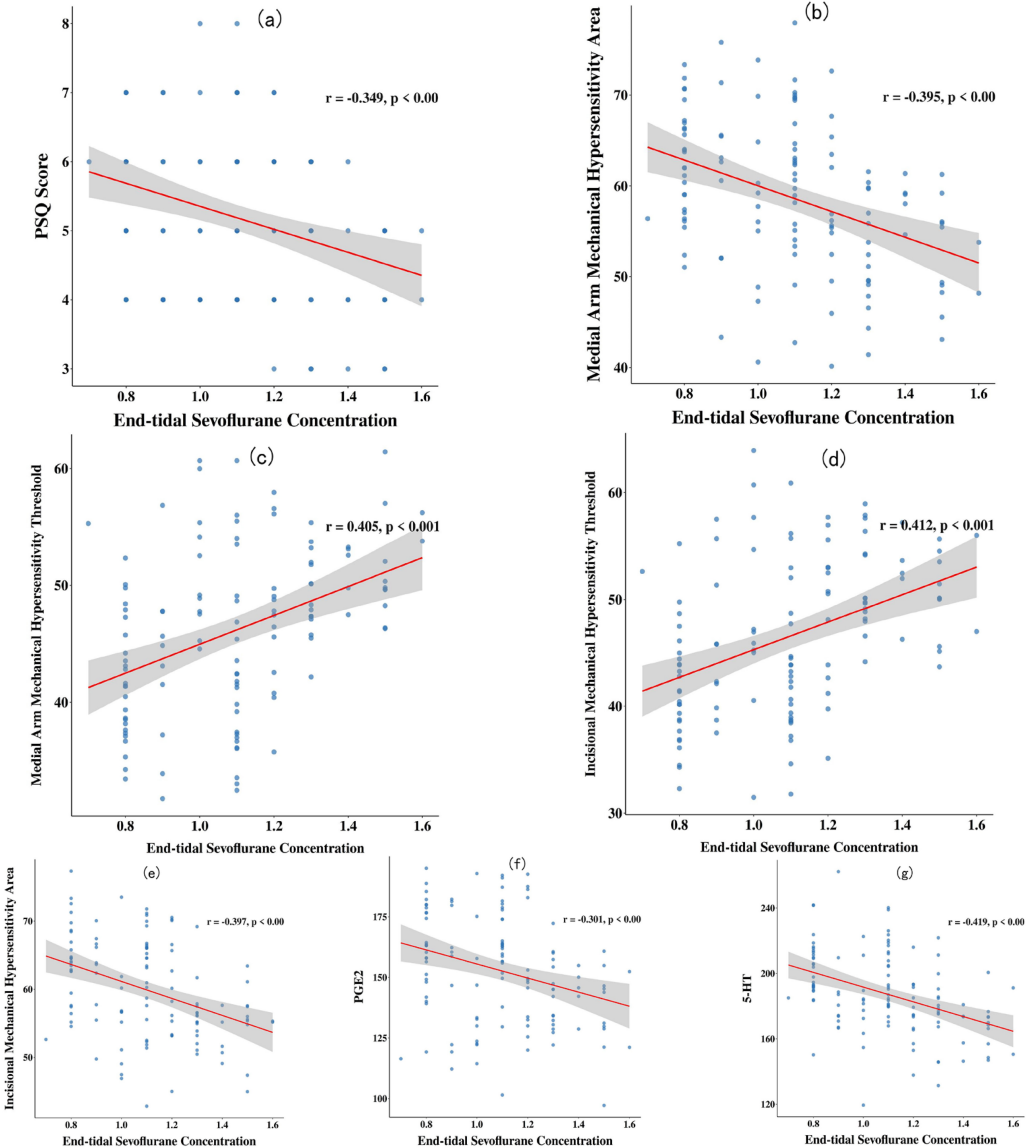
Scatter plots of correlations between end-tidal sevoflurane concentration and pain sensitivity/pain mediators. **(a)** End-tidal sevoflurane concentration was negatively correlated with the PSQ score on postoperative day 1 (*r* = -0.349, *P* < 0.001); **(b)** end-tidal sevoflurane concentration was negatively correlated with the medial arm mechanical hypersensitivity area on postoperative day 1 (*r* = -0.395, *P* < 0.001); **(c)** end-tidal sevoflurane concentration was positively correlated with the medial arm mechanical hypersensitivity threshold on postoperative day 1 (*r* = 0.405, *P* < 0.001); **(d)** end-tidal sevoflurane concentration was positively correlated with the incisional mechanical hypersensitivity threshold on postoperative day 1 (*r* = 0.412, *P* < 0.001); **(e)** end-tidal sevoflurane concentration was negatively correlated with the incisional mechanical hypersensitivity area on postoperative day 1 (*r* = -0.397, *P* < 0.001); **(f)** a higher end-tidal sevoflurane concentration was associated with a smaller area of mechanical hypersensitivity at the incision site on day 1 after surgery (*r* = -0.301, *P* < 0.001); **(g)** end-tidal sevoflurane concentration was negatively correlated with the serum 5-HT level on postoperative day 1 (*r* = -0.419, *P* < 0.001).

Pearson correlation analysis revealed that the end-tidal sevoflurane concentration was positively correlated with the MMSE score, medial arm mechanical hypersensitivity threshold, incisional mechanical hypersensitivity threshold, and NGF levels on postoperative day 1 (*r* > 0, *P* < 0.05). It was negatively correlated with the PSQ score, medial arm mechanical hypersensitivity area, incisional mechanical hypersensitivity area, and serum levels of NSE, ApoJ, PGE2, and 5-HT (*r* < 0, *P* < 0.05) (see Figures 2, 3).

### 3.5 Univariate and multivariate analysis of factors influencing postoperative POCD in elderly patients under general anesthesia

The POCD group had a significantly higher age and higher preoperative day 1 levels of NSE, ApoJ, PGE2, and 5-HT, while the proportion receiving high sevoflurane concentration and preoperative day 1 NGF levels were significantly lower compared to the non-POCD group (*P* < 0.05). Using the occurrence of POCD (Yes = 1, No = 0) as the dependent variable and the indicators showing statistical significance in Table 4 as independent variables (Sevoflurane concentration: High = 0, Low = 1; all other indicators were continuous variables entered as their original values), a multivariate logistic regression model was established. The analysis revealed that increased age and elevated preoperative day 1 levels of NSE, ApoJ, PGE2, and 5-HT were independent risk factors for POCD occurrence (OR > 1, *P* < 0.05). In contrast, high sevoflurane concentration and elevated preoperative day 1 NGF levels were identified as protective factors (OR < 1, *P* < 0.05) (see Tables 3, 4).

**TABLE 3:** Univariate analysis of factors influencing postoperative POCD occurrence in elderly patients under general anesthesia.

Factor	Non-POCD group (*n* = 100)	POCD group (*n* = 21)	χ ^2^/*t*	*P*
Age (years)	68.52 ± 5.16	72.25 ± 4.90	3.037	0.003
Gender (male/female)	53/47	9/12	0.715	0.398
BMI (kg/m^2^)	23.28 ± 2.61	23.92 ± 2.85	1.005	0.317
Hypertension (yes/no)	44/56	13/8	2.233	0.135
Diabetes (yes/no)	37/63	11/10	1.716	0.190
ASA classification (I/II)	25/75	7/14	0.620	0.431
Sevoflurane (high/low)	54/46	6/15	4.489	0.034
Surgery type (gastric/colorectal/hepatobiliary)	39/35/26	9/9/3	1.353	0.508
Anesthesia time (min)	125.26 ± 16.35	127.16 ± 15.85	0.487	0.627
Preoperative day 1 levels of NSE (μg/L)	3.16 ± 0.52	4.89 ± 0.65	13.248	< 0.001
Preoperative day 1 levels of ApoJ (mg/L)	102.36 ± 15.52	131.74 ± 14.85	7.943	< 0.001
Preoperative day 1 levels of NGF (μg/L)	432.56 ± 35.84	381.49 ± 39.45	5.834	< 0.001
Preoperative day 1 levels of PGE2 (pg/mL)	80.26 ± 11.25	93.65 ± 8.49	5.148	< 0.001
Preoperative day 1 levels of 5-HT (pg/mL)	102.36 ± 14.68	139.59 ± 16.28	10.367	< 0.001

**TABLE 4:** Multivariate analysis of factors influencing postoperative POCD occurrence in elderly patients under general anesthesia.

Factor	β	SE	Wald χ ^2^	*P*	OR	95%CI
Age	0.181	0.063	8.246	0.004	1.198	1.059–1.356
Sevoflurane (high)	–1.705	0.601	8.047	0.005	0.182	0.056–0.590
Preoperative day 1 levels of NSE	0.845	0.295	8.205	0.004	2.327	1.306–4.147
Preoperative day 1 levels of ApoJ	0.043	0.015	8.382	0.004	1.044	1.014–1.075
Preoperative day 1 levels of NGF	–0.022	0.007	9.428	0.002	0.978	0.965–0.992
Preoperative day 1 levels of PGE2	0.061	0.025	5.956	0.015	1.063	1.012–1.116
Preoperative day 1 levels of 5-HT	0.031	0.014	4.909	0.027	1.031	1.003–1.060
Constant	–9.451	2.941	10.326	0.001	–	–

## 4 Discussion

POCD primarily manifests as decline in central nervous system functions such as attention, executive function, and memory. Its pathogenesis remains incompletely understood but is considered to be primarily associated with factors such as localized hypoxia, surgical trauma, pain, and imbalances in neurotransmitters (Varpaei et al., 2024). An a large-scale meta-analysis involving 12,921 elderly non-cardiac surgery patients, the incidence of POCD demonstrated a declining trend over time, with rates of 23% at 1 week, 16% at 1 month, and 10% at 3 months postoperatively, the incidence of POCD on day 7 was significantly higher after abdominal surgery compared to orthopedic surgery (β = 0.13, 95% CI: 0.03–0.22, *P* = 0.01) (Huang et al., 2025). Therefore, perioperative cognitive management in elderly patients undergoing abdominal surgery, as a high-risk population for POCD, has become a significant challenge and a key research focus in the field of perioperative medicine. This study administered sevoflurane to elderly patients undergoing general anesthesia for abdominal surgery, observing the effects of different sevoflurane concentrations on cognitive function and pain sensitivity, aiming to provide targeted evidence-based guidance for clinical anesthetic management.

Sevoflurane is an inhalational anesthetic agent, and its safety profile and impact on cognitive function in elderly patients remain subjects of debate (Zhao et al., 2022). An animal study demonstrated that repeated sevoflurane exposure in rats disrupts the tPA/PAI-1 fibrinolytic system balance, thereby impeding the conversion of proBDNF to mature BDNF, low BDNF expression subsequently inhibits the activation of the downstream tropomyosin-related kinase B (TrkB) signaling pathway and reduces hippocampal synaptic plasticity, thereby inducing POCD (Dong et al., 2020). Another study involving 192 patients undergoing laparoscopic surgery maintained anesthesia with inhaled sevoflurane combined with remifentanil. The results showed that on the day of surgery, the mean MMSE score in Group II (40 < BIS ≤ 50) was 29.00 ± 0.89, significantly higher than the 28.336 ± 1.42 in Group I (30 < BIS ≤ 40), indicating that a relatively deeper level of anesthesia had a milder impact on postoperative cognitive function (Shu et al., 2015). The present study found that while the high-concentration group had longer emergence time, time to orientation recovery, spontaneous breathing recovery time, and extubation time compared to the low-concentration group, they also exhibited higher MMSE scores on postoperative days 1 and 3, and a lower incidence of POCD. This suggests that a higher concentration of sevoflurane, despite prolonging recovery times, can mitigate the suppressive effect of general anesthesia on cognitive function and reduce the occurrence of POCD. Serum levels of NSE and ApoJ increased and NGF decreased on postoperative day 1 in both groups compared to their preoperative baselines. However, the high-concentration group demonstrated lower postoperative serum NSE and ApoJ levels, and higher NGF levels, than the low-concentration group. This indicates that while general anesthesia for abdominal surgery causes some degree of neurological impairment, a higher concentration of sevoflurane plays a positive role in alleviating neuronal damage and promoting neural repair. The potential reasons are hypothesized as follows: (1) Sevoflurane pretreatment or exposure might upregulate the expression of superoxide dismutase and glutathione peroxidase by activating the nuclear factor erythroid 2-related factor 2 (Nrf2) signaling pathway, thereby enhancing antioxidant enzyme activity, mitigating oxidative stress induced by surgical trauma or the anesthetic itself, providing neuronal protection, and consequently reducing the risk of POCD (Varpaei et al., 2024; Zhou et al., 2016). (2) Sevoflurane can inhibit the activation of nuclear factor kappa B (NF-κB), downregulate the expression of NF-κB-dependent inflammatory genes, suppress the release of inflammatory mediators, and thus alleviate inflammation-related neurological damage (Peipei et al., 2022). Pearson correlation analysis in this study revealed that the end-tidal sevoflurane concentration was positively correlated with the MMSE score and NGF levels, and negatively correlated with NSE and ApoJ levels on postoperative day 1. Furthermore, a high sevoflurane concentration [OR: 0.182 (95% CI: 0.056–0.590)] was identified as a protective factor against POCD in elderly patients undergoing abdominal surgery under general anesthesia. This further confirms the close relationship between sevoflurane concentration and postoperative cognitive function, suggesting that appropriately increasing the sevoflurane concentration might be a feasible neuroprotective strategy. In this study, remifentanil dosage in both groups was adjusted based on BIS and MAP fluctuations, and a standardized PCIA regimen was used postoperatively. Statistical analysis confirmed no significant difference in remifentanil consumption between the groups. Furthermore, patients using benzodiazepines, ketamine, or similar medications preoperatively were excluded, and these drugs were not administered intraoperatively. This suggests that differences in analgesic medication use were not the primary factor contributing to the observed POCD differences between the groups.

This study found that the high-concentration group exhibited lower PSQ scores, smaller medial arm and incisional mechanical hypersensitivity areas, and higher medial arm and incisional mechanical hypersensitivity thresholds on postoperative day 1 compared to the low-concentration group. Furthermore, the end-tidal sevoflurane concentration was significantly correlated with the PSQ score, mechanical hypersensitivity thresholds, and mechanical hypersensitivity areas. These results indicate that a higher concentration of sevoflurane helps reduce postoperative pain sensitivity and mechanical hypersensitivity areas while elevating pain thresholds in patients, suggesting it plays an important role in inhibiting hyperalgesia and central sensitization. The underlying mechanisms may involve the following aspects: On one hand, sevoflurane can act directly on the central nervous system, modulating N-methyl-D-aspartate (NMDA) receptor activity and interfering with pain signal transmission (Guo et al., 2024; Xie et al., 2024). On the other hand, data from this study demonstrate that a higher concentration of sevoflurane suppresses the postoperative release of pain mediators (PGE2, 5-HT), thereby alleviating pain sensitivity.

Painful stress promotes excessive activation of the hypothalamic-pituitary-adrenal (HPA) axis, leading to elevated cortisol levels. Excessively high cortisol levels exert toxic effects on hippocampal neurons, thereby impairing learning ability and spatial memory (Cao et al., 2024). Furthermore, pain promotes overactivation of central nervous cells and the release of large quantities of inflammatory factors, exacerbates sympathetic nerve excitability, and impairs the capacity for information exchange between brain cells. This consequently leads to cerebral hypoxia-ischemia and disruption of the cerebral microcirculation (Irvine and Clark, 2018). The multivariate logistic regression model established in this study identified elevated preoperative day 1 levels of PGE2 and 5-HT as risk factors for POCD in elderly patients after general anesthesia, supporting the aforementioned theory. Additionally, a domestic study reported that the PSQ score correlates with postoperative pain intensity, and pain sensitivity can effectively predict the occurrence of chronic postoperative pain and neuropathic pain (Wu et al., 2023). This demonstrates that pain sensitivity indirectly influences patients’ cognitive function. In this study, the high sevoflurane concentration reduced pain sensitivity and mechanical hypersensitivity in elderly patients undergoing abdominal surgery under general anesthesia, thereby interrupting the pathological process of “pain-stress-cognitive impairment” and consequently exerting a neuroprotective function after general anesthesia.

## 5 Conclusion

The concentration of sevoflurane during general anesthesia in elderly patients is closely associated with postoperative cognitive function and pain sensitivity. A higher sevoflurane concentration can reduce pain sensitivity, inhibit the expression of pain mediators and neurological damage markers, alleviate cognitive impairment, and serves as a protective factor against the development of POCD. Several limitations must be acknowledged. Firstly, the single-center observational design and limited sample size may introduce confounding biases. Consequently, our results require confirmation from future multi-center, large-sample randomized controlled trials. This study did not include other inhalational or intravenous anesthetics as controls, making it difficult to entirely exclude the potential for cross-drug interactions. Furthermore, the incidence of POCD was only recorded up to postoperative day 3; it is recommended that future studies extend the follow-up period to observe the medium- and long-term effects of sevoflurane on cognitive function in elderly patients after general anesthesia. Additionally, as an observational association study, while correlations were established, causality cannot be definitively determined. ASA III elderly patients are common in clinical practice. However, due to their reduced organ functional reserve and higher burden of comorbidities, their anesthesia tolerance and postoperative recovery characteristics differ from those of ASA I-II patients. The impact of sevoflurane concentration on POCD and pain sensitivity in this specific population remains unclear, which may limit the generalizability of our findings. Future studies specifically targeting ASA III elderly patients are needed to further validate the clinical application value of sevoflurane concentration. The specific mechanisms through which sevoflurane influences cognitive function and pain sensitivity were not thoroughly investigated; future studies incorporating basic science experiments are needed to further elucidate the precise mechanisms of action at the signaling pathway and molecular levels.

## Data Availability

The datasets generated and analyzed during the current study are not publicly available due to institutional restrictions but are available from the corresponding author upon request.

## References

[B1] Apfelbaum J. L. Connis R. T. (2019). The American society of anesthesiologists practice parameter methodology. *Anesthesiology* 130 367–384. 10.1097/ALN.0000000000002551 30724774

[B2] Cao L. Ye S. Chen Y. Pei Y. Chen J. Li X. (2024). Longitudinal study on the trajectory and influencing factors of cognitive dysfunction in lung transplantation patients. *Transpl. Immunol.* 84:102053. 10.1016/j.trim.2024.102053 38750974

[B3] Cao S. J. Zhang Y. Zhang Y. X. Zhao W. Pan L. H. Sun X. D. (2023). Delirium in older patients given propofol or sevoflurane anaesthesia for major cancer surgery: A multicentre randomised trial. *Br. J. Anaesth.* 131 253–265. 10.1016/j.bja.2023.04.024 37474241

[B4] Dong Y. Hong W. Tang Z. Gao Y. Wu X. Liu H. (2020). Sevoflurane leads to learning and memory dysfunction via breaking the balance of tPA/PAI-1. *Neurochem. Int.* 139:104789. 10.1016/j.neuint.2020.104789 32650025

[B5] Guo F. Zhang B. Shen F. Li Q. Song Y. Li T. (2024). Sevoflurane acts as an antidepressant by suppression of GluN2D-containing NMDA receptors on interneurons. *Br. J. Pharmacol.* 181 3483–3502. 10.1111/bph.16420 38779864

[B6] Hu N. Guo D. Wang H. Xie K. Wang C. Li Y. (2014). Involvement of the blood-brain barrier opening in cognitive decline in aged rats following orthopedic surgery and high concentration of sevoflurane inhalation. *Brain Res.* 1551 13–24. 10.1016/j.brainres.2014.01.015 24440777

[B7] Huang W. W. Y. Fan S. Li W. Y. Thangavelu V. Saripella A. Englesakis M. (2025). Prevalence of postoperative neurocognitive disorders in older non-cardiac surgical patients: A systematic review and meta-analysis. *J. Clin. Anesth.* 103:111830. 10.1016/j.jclinane.2025.111830 40199029

[B8] Irvine K. A. Clark J. D. (2018). Chronic pain after traumatic brain injury: Pathophysiology and pain mechanisms. *Pain Med.* 19 1315–1333. 10.1093/pm/pnx153 29025157

[B9] Kitthanyateerakul P. Tankumpuan T. Davidson P. M. (2024). Cognitive dysfunction in older patients undergoing non-neurosurgery in the immediate postoperative period: A systematic review. *Nurs. Open* 11:e70023. 10.1002/nop2.70023 39189543 PMC11348231

[B10] Laferrière-Langlois P. Morisson L. Jeffries S. Duclos C. Espitalier F. Richebé P. (2024). Depth of anesthesia and nociception monitoring: Current state and vision for 2050. *Anesth. Analg.* 138 295–307. 10.1213/ANE.0000000000006860 38215709

[B11] Li H. Jia J. Yang Z. (2016). Mini-Mental state examination in elderly chinese: A population-based normative study. *J. Alzheimers Dis.* 53 487–496. 10.3233/JAD-160119 27163822

[B12] Peipei W. Ping W. Miaomiao Y. Shuo W. (2022). Sevoflurane ameliorates LPS-induced inflammatory injury of HK-2 cells through Sirtuin1/NF-κB pathway. *Allergol. Immunopathol.* 50 115–123. 10.15586/aei.v50i4.623 35789410

[B13] Quan X. Fong D. Y. T. Leung A. Y. M. Liao Q. Ruscheweyh R. Chau P. H. (2018). Validation of the Mandarin Chinese Version of the pain sensitivity questionnaire. *Pain Pract.* 18 180–193. 10.1111/papr.12587 28422444

[B14] Shu A. H. Wang Q. Chen X. B. (2015). Effect of different depths of anesthesia on postoperative cognitive function in laparoscopic patients: A randomized clinical trial. *Curr. Med. Res. Opin.* 31 1883–1887. 10.1185/03007995.2015.1075968 26202165

[B15] Suraarunsumrit P. Srinonprasert V. Kongmalai T. Suratewat S. Chaikledkaew U. Rattanasiri S. (2024). Outcomes associated with postoperative cognitive dysfunction: A systematic review and meta-analysis. *Age Ageing* 53:afae160. 10.1093/ageing/afae160 39058915 PMC11277860

[B16] Varpaei H. A. Farhadi K. Mohammadi M. Khafaee Pour Khamseh A. Mokhtari T. (2024). Postoperative cognitive dysfunction: A concept analysis. *Aging Clin. Exp. Res.* 36:133. 10.1007/s40520-024-02779-7 38902462 PMC11189971

[B17] Viderman D. Nabidollayeva F. Aubakirova M. Yessimova D. Badenes R. Abdildin Y. (2023). Postoperative delirium and cognitive dysfunction after general and regional Anesthesia: A systematic review and meta-analysis. *J. Clin. Med.* 12:3549. 10.3390/jcm12103549 37240655 PMC10219265

[B18] Wu Q. Luo Y. Han M. Li J. Kang F. (2023). The value of pain sensitivity questionnaire in predicting postoperative pain in living kidney donors: A prospective observational study. *J. Pain Res.* 16 2899–2907. 10.2147/JPR.S419719 37641638 PMC10460613

[B19] Xie A. Zhang X. Ju F. Zhou Y. Wu D. Han J. (2024). Sevoflurane impedes neuropathic pain by maintaining endoplasmic reticulum stress and oxidative stress homeostasis through inhibiting the activation of the PLCγ/CaMKII/IP3R signaling pathway. *Aging* 16 11062–11071. 10.18632/aging.206001 38975935 PMC11272110

[B20] Xu J. H. Zhang T. Z. Peng X. F. Jin C. J. Zhou J. Zhang Y. N. (2013). Effects of sevoflurane before cardiopulmonary bypass on cerebral oxygen balance and early postoperative cognitive dysfunction. *Neurol. Sci.* 34 2123–2129. 10.1007/s10072-013-1347-3 23525738

[B21] Yang N. S. Zhong W. J. Sha H. X. Zhang C. Y. Jin L. Duan J. X. (2024). mtDNA-cGAS-STING axis-dependent NLRP3 inflammasome activation contributes to postoperative cognitive dysfunction induced by sevoflurane in mice. *Int. J. Biol. Sci.* 20 1927–1946. 10.7150/ijbs.91543 38481801 PMC10929193

[B22] Zeng K. Long J. Li Y. Hu J. (2023). Preventing postoperative cognitive dysfunction using anesthetic drugs in elderly patients undergoing noncardiac surgery: A systematic review and meta-analysis. *Int. J. Surg.* 109 21–31. 10.1097/JS9.0000000000000001 36799783 PMC10389238

[B23] Zhao D. Zhang M. Yang L. Zeng M. (2022). GPR68 improves nerve damage and myelination in an immature rat model induced by sevoflurane anesthesia by activating cAMP/CREB to mediate BDNF. *ACS Chem. Neurosci.* 13 423–431. 10.1021/acschemneuro.1c00830 35025202

[B24] Zhou Z. B. Yang X. Y. Tang Y. Zhou X. Zhou L. H. Feng X. (2016). Subclinical concentrations of sevoflurane reduce oxidative stress but do not prevent hippocampal apoptosis. *Mol. Med. Rep.* 14 721–727. 10.3892/mmr.2016.5336 27222114 PMC4918604

[B25] Zhu J. Chen C. Wu J. He M. Li S. Fang Y. (2023). Effects of propofol and sevoflurane on social and anxiety-related behaviours in sleep-deprived rats. *Br. J. Anaesth.* 131 531–541. 10.1016/j.bja.2023.05.025 37543435

